# Needle-knife lumbosacral therapy for lumbar disc herniation: a single-center, outcome assessor-blinded randomized controlled trial

**DOI:** 10.3389/fneur.2026.1898714

**Published:** 2026-10-16

**Authors:** Yiming Zhang, Ruiguo Li, Xiaohong Zhang, Zhanyou Wang, Ziwei Zhang, Huanhuan Xu, Xiaofan Li

**Affiliations:** 1 College of Acupuncture and Massage, Henan University of Chinese Medicine, Zhengzhou, China; 2 The Third Affiliated Hospital of Henan University of Chinese Medicine, Zhengzhou, China

**Keywords:** clinical observation, controlled trial, lumbar disc herniation, lumbosacral co-treatment, needle-knife

## Abstract

**Background:**

Lumbar disc herniation (LDH) is a common spinal disorder causing significant pain and dysfunction. Traditional acupuncture is a widely used non-pharmacological treatment for LDH. Recently, needle-knife therapy has emerged as a potential alternative.

**Objective:**

This study aims to explore the clinical efficacy of needle-knife lumbosacral therapy for LDH and compare its advantages with those of conventional acupuncture therapy.

**Methods:**

This study adopted an outcome assessor-blinded randomized controlled trial design. A total of 74 patients with LDH were enrolled and randomly assigned to the experimental group (*n* = 37), which received needle-knife lumbosacral therapy, or the control group (*n* = 37), which received conventional acupuncture at the same anatomical treatment sites. Seventy patients completed the 2-week treatment and 3-month follow-up. Visual analog scale (VAS) and Oswestry Disability Index (ODI) were designated as primary repeated outcomes; lumbar flexion mobility (LFM) was a secondary repeated outcome, and inflammatory cytokines served as non-repeated secondary outcomes. Mixed-effects linear models were used for repeated measurements, with the group-by-time interaction predefined as the primary estimand. Bonferroni correction was applied for the three repeated outcomes (VAS, ODI, and three LFM components), setting an adjusted significance threshold of *α* = 0.017. Unadjusted *α* = 0.05 was adopted for inflammatory cytokines. VAS, ODI, and LFM were evaluated at baseline, post-treatment, and 3-month follow-up; inflammatory markers (TNF-α, IL-6, IL-1β) were detected at baseline and post-treatment only. Adverse events were monitored throughout the trial. Patient recruitment was conducted from March 15, 2025, to July 15, 2025, and the final follow-up was performed on November 18, 2025.

**Results:**

Both groups demonstrated within-group reductions in VAS and ODI scores across all time points. For the prespecified group-by-time interaction analyses under Bonferroni correction (*α* = 0.017), the needle-knife group showed significantly larger longitudinal improvements than the acupuncture group for both primary outcomes: VAS interaction (95% CI: 0.21–0.83), adjusted *p* = 0.001; ODI interaction (95% CI: 1.89–4.35), adjusted *p* < 0.0001. At post-treatment, the model-based between-group differences (needle-knife minus acupuncture) were VAS: −1.16 (95% CI: −1.78 to −0.54), adjusted *p* = 0.0004; ODI: −3.78 (95% CI: −4.95 to −2.61), adjusted *p* < 0.0001. At 3-month follow-up, sustained between-group differences were VAS: −0.43 (95% CI: −0.68 to −0.18), adjusted *p* = 0.001; ODI: −1.89 (95% CI: −2.56 to −1.22), adjusted *p* < 0.0001. For LFM components, all three subindices showed greater numerical improvements in the needle-knife group, but their group-by-time interaction adjusted *p*-values exceeded the 0.017 threshold: lumbar flexion (95% CI: 0.62–5.00), adjusted *p* = 0.034; left lateral flexion (95% CI: −1.02 to 2.42), adjusted *p* = 0.423; right lateral flexion (95% CI: −1.10 to 1.92), adjusted *p* = 0.594. Among serum inflammatory biomarkers, only IL-6 showed a statistically significant unadjusted between-group difference at post-treatment: IL-6 difference = −0.47 ng·L (95% CI: −0.89 to −0.05), unadjusted *p* = 0.03; TNF-α difference = −0.18 ng·L (95% CI: −0.52 to 0.16), unadjusted *p* = 0.30; IL-1β difference = −0.13 ng·L (95% CI: −0.37 to 0.11), unadjusted *p* = 0.28. No serious adverse events were reported.

**Conclusion:**

Two-week lumbosacral needle-knife therapy effectively relieves pain and lumbar dysfunction in LDH patients, with statistically verified sustained superiority over conventional acupuncture for VAS and ODI under the Bonferroni-adjusted threshold (*α* = 0.017). Although numerical advantages in lumbar mobility were observed in the needle-knife group, these differences did not reach the prespecified adjusted significance level after multiple testing correction. As an outcome assessor-blinded exploratory trial with unmasked participants and therapists, large-sample and multicenter clinical studies are needed to further verify the conclusions, and future trials should optimize blinding schemes to reduce reporting bias.

**Clinical trial registration:**

https://itmctr.ccebtcm.org.cn/mgt/project/user/approved-list, identifier (ITMCTR2025002191).

## Introduction

Lumbar disc herniation (LDH) is a prevalent clinical condition, with global epidemiological studies reporting an annual incidence of approximately 2–3% and a lifetime risk of 5–10%. It exhibits a male-to-female ratio of about 7:1 and most commonly affects individuals aged 30–50 years (1). Based on the extent of nucleus pulposus protrusion, LDH is categorized into bulging, protrusion, and extrusion types (Figure 1) (2). The fundamental pathogenesis of LDH involves intervertebral disc degeneration and mechanical stress (3). With advancing age, the nucleus pulposus loses proteoglycans and water, diminishing its elasticity. Concurrently, the annulus fibrosus also undergoes changes, including collagen breakdown and fissure formation (4). Under prolonged axial loading, rotational forces, or flexion stress, the degenerated nucleus pulposus herniates through weakened areas of the annulus fibrosus (often posterolaterally), compressing nerve roots or the dural sac (5). This mechanical compression is accompanied by the release of inflammatory mediators such as IL-6, TNF-α, and PGE₂, leading to nerve root ischemia, edema, and pain sensitization (6). Clinical manifestations include low back pain accompanied by radiating lower limb pain, exacerbated by coughing, prolonged sitting, or flexion movements, significantly impairing patients’ daily work and life.

**Figure 1:**
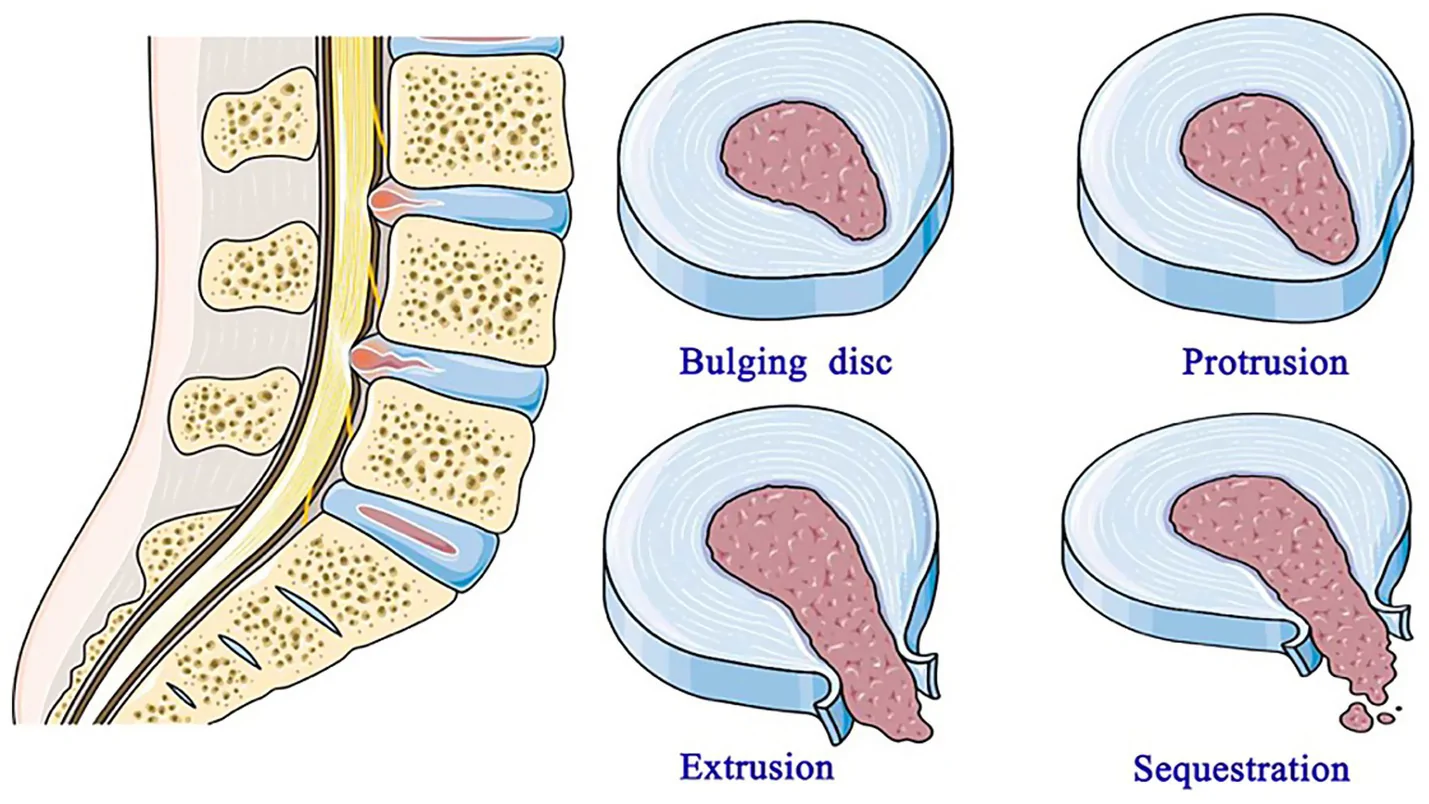
Classification of lumbar disc herniation. Adapted from Servier Medical Art via Bioicons (https://bioicons.com), licensed under CC BY 3.0.

Clinical interventions for LDH are generally divided into conservative and surgical regimens. Conservative modalities encompassing acupuncture, manipulative therapy and oral pharmacotherapy have yielded partial therapeutic benefits yet possess inherent drawbacks, including prolonged therapeutic courses, incapability to directly decompress mechanically compressed nerve roots, and a high recurrence risk upon treatment withdrawal (7).

Surgical decompression serves as an alternative therapeutic option capable of rapidly releasing radicular compression and promptly ameliorating clinical manifestations. However, invasive surgical procedures are accompanied by unavoidable operative risks, among which iatrogenic neurological injury stands as a adverse event. Against the backdrop of the limitations inherent to conservative protocols and the neurological complications associated with surgical management, Western clinical researchers are increasingly dedicated to developing innovative therapeutic interventions characterized by high clinical operability and negligible adverse reactions (8).

Needle-knife therapy evolved from traditional acupuncture therapy, compensating for the limitation of conventional acupuncture in effectively releasing the tension of soft tissue surrounding the spine (9). A recent clinical study demonstrated that Needle-knife therapy significantly reduces VAS and ODI scores in LDH patients, suggesting superior efficacy compared to traditional acupuncture (10). The sacroiliac joint serves as a foundational component in the spine–pelvis–lower limb mechanical chain (11). During the acute phase of LDH, approximately 68–85% of patients develop sacroiliac joint dysfunction, resulting in the persistence and chronicity of low back pain symptoms (12). Thus, treatment of the sacroiliac joint should be prioritized in LDH therapy.

Needle-knife lumbosacral therapy is defined as unified minimally invasive release of lumbosacral paravertebral and sacroiliac soft tissues, distinct from single lumbar-site needle-knife release described in previous literature. Full acupoint layout and manipulation details are provided in Methods (Figure 2 and Table 1).

**Figure 2:**
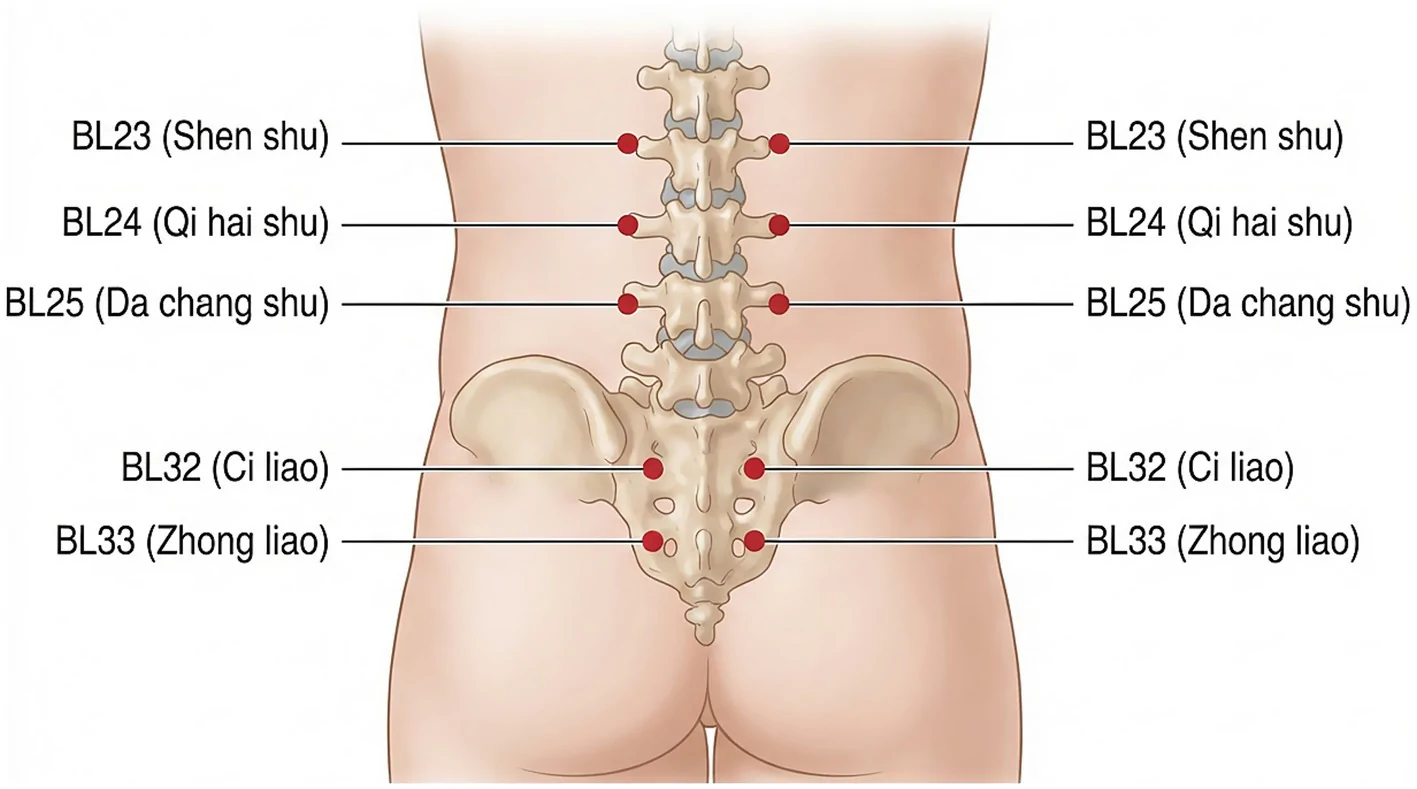
The specific anatomical locations of the acupoints for acupuncture and needle-knife therapy.

**Table 1:** Needle-knife instruments and release methods.

Acupoint	Needle-knife instrument	Needle-knife operation method
Qi hai shu (BL21)	1.0 mm × 80 mm	The three-step slow push method^a^
Da chang shu (BL25)	1.0 mm × 80 mm	The three-step slow push method
Shen shu (BL23)	1.0 mm × 80 mm	The three-step slow push method
Ci liao (BL32)	1.0 mm × 80 mm	The three-step slow push method
Zhong liao (BL33)	1.0 mm × 80 mm	The three-step slow push method

a The operation method of the needle knife is as follows.

1. The physician locates the point with the left hand and holds the needle knife with the right hand, inserting the needle from the outer side of the fingertip.

2. Before needle insertion, the patient is instructed to take a deep breath. The physician swiftly inserts the needle-knife along the outer edge of the supporting finger into the subcutaneous tissue.

3. A brief pause of 3–5 s follows, during which the patient is guided to breathe deeply and slowly. As the patient’s exhalation is about to end, longitudinal cutting and transverse stripping are performed 2–3 times to release the superficial tissues.

4. After another pause of 3–5 s, the patient is again guided to breathe deeply and slowly. As the exhalation is about to end, the needle knife is gently advanced into the middle layer. Longitudinal cutting and transverse stripping are performed 2–3 times to release the middle layer soft tissues.

5. Following a pause of 3–5 s to confirm the accuracy of the treatment site, the patient is guided to breathe deeply and slowly. As the exhalation is about to end, the needle knife is gently advanced into the deep layer until the bone surface is reached. Longitudinal cutting and transverse stripping are performed 2–3 times.

6. After a final pause of 3–5 s, the patient’s sensation is inquired about. The patient is guided to breathe deeply and slowly, and as the patient inhales, the needle knife is quickly withdrawn. After needle withdrawal, the therapy site is compressed for 15 min to stop bleeding, covered with a sterile gauze, and kept dry for 24 h.

Based on these considerations, we put forward a single directional primary hypothesis: after a 2-week standardized treatment course, both needle-nife lumbosacral therapy and conventional acupuncture can reduce patients’ VAS scores and ODI scores, while patients receiving needle-knife lumbosacral therapy will achieve significantly greater declines in VAS and ODI from baseline to the immediate post-treatment timepoint and maintain superior improvements at the 3-month follow-up compared with those treated with conventional acupuncture; all assessments of lumbar flexion mobility, serum inflammatory cytokines, and adverse event profiles are defined as exploratory secondary analyses rather than confirmatory tests, which is the core premise for us to conduct this single-blind randomized controlled trial to compare the clinical advantages of needle-knife lumbosacral therapy versus conventional acupuncture for lumbar disc herniation.

## Method

### Study design

This study adopted an outcome assessor-blinded randomized controlled trial design. Marked disparities exist between needle-knife therapy and conventional acupuncture in operating tools, soft tissue cutting-stripping manipulation, stimulation depth and sensory experience. Moreover, 0.5% lidocaine local infiltration anesthesia was exclusively used in the needle-knife experimental group, while no anesthetic intervention was provided for the acupuncture control group. These inherent differences prevented full masking of participants and treating therapists, both of whom could clearly distinguish their assigned intervention. To reduce evaluation bias, only independent outcome assessors who measured and recorded all primary and secondary indicators were blinded to group allocation throughout the trial. Participants with LDH were randomly allocated at a 1:1 ratio into either an experimental group or a control group using a random number table method. Both groups received standard treatment (ST). The experimental group received needle-knife lumbosacral therapy (NKT), while the control group received acupuncture therapy (AT). The trial protocol was approved by the Ethics Committee of Henan University of Chinese Medicine (Approval No: 2025HL-003) and monitored by a safety oversight committee. This prospective randomized controlled trial focusing on patients with lumbar disc herniation was carried out within the framework of an ethically approved parent research project concerning chronic nonspecific low back pain. The complete study protocol is available in Supplementary material 1. All participants provided written informed consent under the principles of the Declaration of Helsinki (13). The trial was designed and reported in accordance with the Consolidated Standards of Reporting Trials [CONSORT (14)] guidelines and the Standards for Reporting Interventions in Clinical Trials of Acupuncture [STRICTA (15)]. The study was registered with the International Traditional Medicine Clinical Trial Registration Platform (Registration ID: ITMCTR2025002191), and the protocol has been made publicly accessible. At enrolment, baseline demographic and clinical characteristics were collected for all participants, including age, sex, duration of symptoms, body mass index (BMI), and smoking status. BMI and smoking status are recognised risk factors associated with the onset and progression of lumbar disc herniation.

### Participants

Patients from the Acupuncture Department of the Third Affiliated Hospital of Henan University of Chinese Medicine were screened according to the following inclusion and exclusion criteria. Inclusion Criteria: 1. Patients who met the diagnostic criteria for LDH in the “Diagnostic Criteria for Traditional Chinese Medicine Diseases” in China (low back pain accompanied by radiating leg pain). 2. Diagnosed with lumbar disc herniation via combined clinical manifestations and standardized L3–L5 lumbar MRI: consistent radicular lumbocrural pain, positive straight leg raise or Bragard sign, and MRI-verified disc bulging/protrusion/extrusion compressing corresponding nerve roots at L3–L5 segments; individuals with isolated asymptomatic disc changes on MRI without matching radiating limb pain were excluded. 3. Aged between 20 and 65 years old. 4. With symptoms lasting for 3 months or longer. 6. VAS score > 5 points. Exclusion Criteria: 1. Patients with vertebral fractures, spinal infections, or active inflammatory diseases within the past year. 2. Patients who were unable to complete the assessment. 3. Patients suffering from serious diseases (such as cancer, severe cardiovascular diseases like heart failure, advanced liver diseases including liver cirrhosis and liver failure, severe blood diseases like leukemia, and other life-threatening diseases). 4. Pregnant women, women planning to conceive, or lactating women. 5. Patients who had participated in other acupuncture therapies within the past month. Withdrawal Criteria: 1. Patients who did not follow the treatment protocol or received additional treatments not specified in the study during the treatment period. 2. Patients who experienced serious adverse reactions during the treatment process. 3. Patients who were unable to cooperate with the treatment for other reasons and voluntarily withdrew from the study.

### Randomization and blinding

Randomization was performed by a statistician independent of participant recruitment, intervention delivery, and outcome assessment using SPSS Statistics Version 26.0 (IBM). A 1:1 random allocation sequence was generated, printed onto sequentially-numbered, tamper-proof opaque sealed envelopes. All envelopes were centrally locked and stored by an unblinded research coordinator who was not involved in screening or enrolment. Each envelope was opened only after a participant had completed enrolment and provided written informed consent. Recruiting clinicians had no access to unopened envelopes and could not foresee or predict upcoming group assignment. All efficacy assessments were conducted by a clinician unaware of the study design or patient allocation.

### Intervention

All participants in both the experimental and control groups received ST (comprised of controlled management of hypertension and hyperglycemia, supplemented with oral methylcobalamin, 500 μg three times daily). Both groups underwent a systematic treatment course lasting 2 weeks. Acupuncture procedures were performed by a licensed practitioner with over 5 years of clinical experience, while needle-knife therapy was conducted by a chief physician. Prior to the study, all therapists received standardized training focusing on precise localization of acupoints and needle-knife release techniques.

The control group received acupuncture treatment four times per week (Monday–Thursday), with each session lasting 40 min. A complete treatment course consists of 2 weeks. The acupoint prescription was determined based on theories of Traditional Chinese Medicine and previous literature related to acupuncture for LDH (16). The selected treatment sites were the bilateral transverse processes of L3, L4, and L5, corresponding to Shen shu (BL23), Qi hai shu (BL24), Da chang shu (BL25) and the bilateral sacral foramina corresponding to Ci liao (BL32) and Zhong liao (BL33). Acupoint nomenclature and localization followed the National Standard of the People’s Republic of China (GB/T 12346-2006). Detailed information regarding the locations of these acupoints can be found in Figure 2. Sterile, single-use stainless steel acupuncture needles (manufactured by Bangde Medical Equipment Co., Maanshan, China; specifications: 0.30 × 25 mm, 0.30 × 40 mm, and 0.30 × 75 mm) were used, with needle specifications selected according to the thickness of the local soft tissue at each acupoint (25 mm for superficial points, 40 mm for medium-depth points, and 75 mm for deep points). For each acupoint, needling techniques such as lifting and thrusting, twisting, and rotating were applied for 1 min to induce the sensation of deqi, characterized by local soreness, numbness, distension, and pain. After achieving deqi, needles were retained for 30 min. The cumulative number of treated points per session was ten, with a total of 80 points over the entire treatment course.

Participants in the experimental group received needle-knife treatment four times per week (Monday–Thursday). Each treatment session targeted two specific acupoints, which were selected in a rotating sequence to ensure that all five bilateral acupoint pairs (ten points in total) were treated over the course of five sessions. The treatment sequence was standardized as follows: Session 1: BL23 bilaterally; Session 2: BL24 bilaterally; Session 3: BL25 bilaterally; Session 4: BL32 bilaterally; Session 5: BL33 bilaterally; this sequence was then repeated for the remaining three sessions, resulting in all acupoints being treated twice over the two-week course. The entire intervention lasted 2 weeks as one treatment course, totaling eight sessions, with a cumulative total of 16 treated points per participant. The duration of each needle-knife session ranged from 15 to 20 min, depending on the complexity of soft tissue adhesions encountered. The treatment procedures were as follows: 1. Localization: The treatment locations in the experimental group were identical to those in the control group. 2. Patient’s position: Patients were placed in a prone position with the face resting in the face hole of the treatment table to ensure neck relaxation. The upper limbs were positioned alongside the body or allowed to hang freely from both sides of the Table 1. Disinfection: The skin around the treatment site was disinfected using iodophor-saturated cotton swabs, wiping in three concentric circles outward from the treatment point, ensuring no gaps were left. The disinfection area extended radially at least 10 cm from the site. A sterile aperture drape was then applied to expose the target area while maintaining an aseptic field. 4. Anesthesia: Local anesthesia was administered using a 0.5% lidocaine solution. A volume of 1 mL was injected per treatment site. During the procedure, patients were instructed to breathe normally. The needle was advanced slowly until bony contact was achieved. A retrograde injection technique was employed, distributing the 1 mL solution gradually from deep to superficial tissue. 5. The needle-knife therapy was performed using a Zhougong-brand Type I No. 3 needle-knife (1.0 mm × 80 mm). The shape and specification of the needle-knife are shown in Figure 3. The “three-step slow stratified needle insertion technique” (17) was employed to advance the needle-knife gradually to the bony surface. During needle-knife insertion, longitudinal cutting and transverse stripping were performed at the superficial, intermediate, and deep soft tissue layers, with two to three repetitions at each layer. Emphasis was placed on soft tissue adhesions around the spinal vertebrae. A detailed description of the operational method is provided in Table 1. To control for operator-dependent variability, all needle-knife procedures were performed by the same chief physician throughout the study, and the manipulation technique, needling depth, and number of cutting/stripping repetitions were standardized according to the protocol in Table 1.

**Figure 3:**
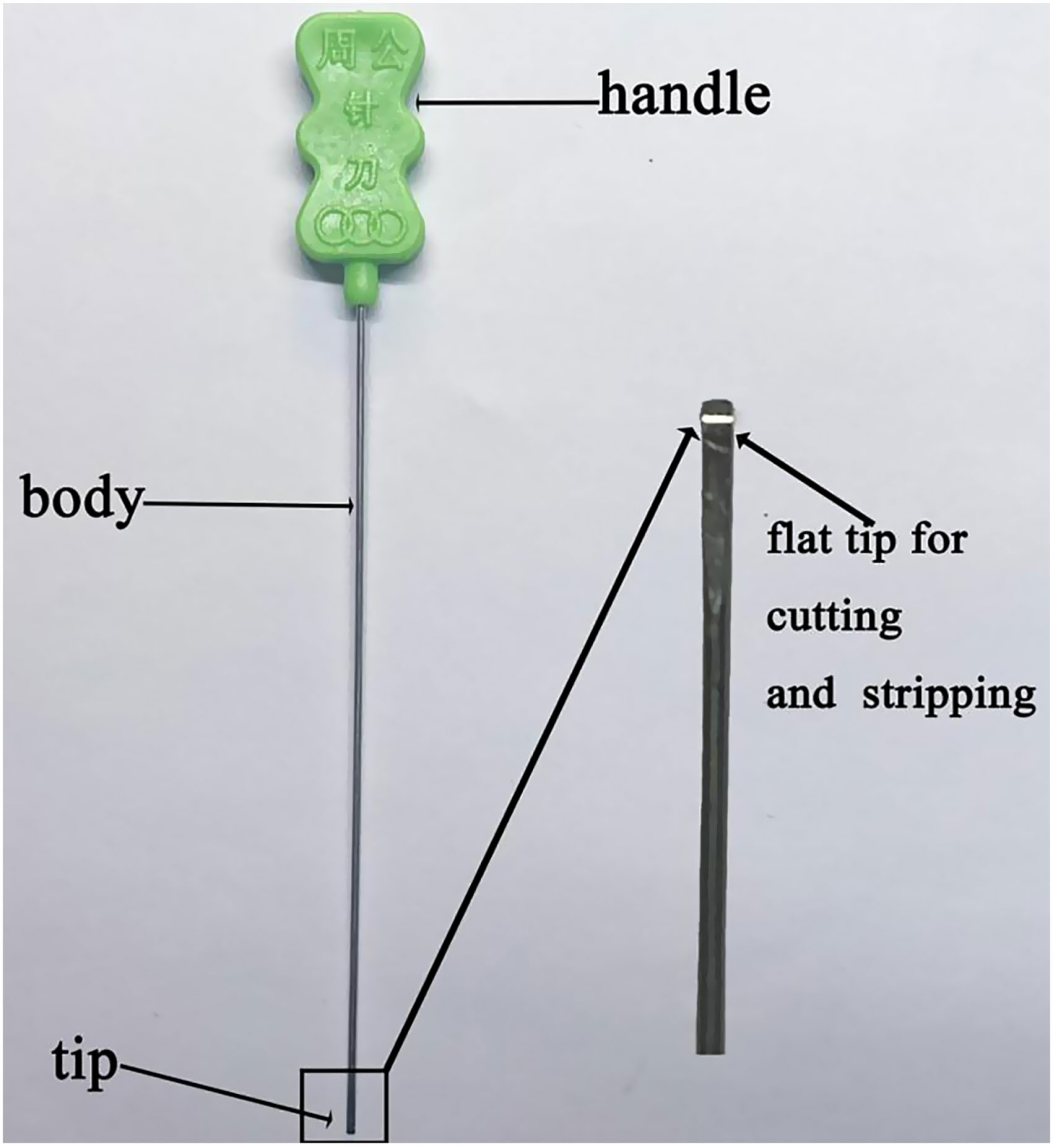
Shape and specification of the needle knife.

It is noteworthy that, in the experimental group, 1 mL of 0.5% lidocaine was administered via local infiltration at each treatment site prior to needle-knife manipulation, whereas no anesthetic or sham injection was given to the control group. This design was implemented primarily to enhance patient tolerance and mitigate procedural pain associated with deep soft tissue penetration and periosteal manipulation, thereby ensuring safe and complete execution of the needle-knife procedure. However, this asymmetry introduces a potential confounding factor, as the between-group comparison encompasses not only the distinct therapeutic modalities but also the analgesic effect of lidocaine, the physiological and psychological responses to the injection itself, and differential procedural exposure. The implications of this design are further addressed in the Discussion section.

To ensure comparability between groups and control for potential operator effects and treatment dose differences, the following measures were implemented: 1. all participants in both groups were treated by the same designated practitioner throughout the entire study period (the chief physician for the needle-knife group and the licensed acupuncturist for the acupuncture group); 2. all treatment sessions were scheduled at the same time of day (between 8:00 a.m. and 10:00 a.m.) to minimize circadian variations in pain perception; 3. the total intervention duration was standardized to 2 weeks for both groups; 4. the cumulative number of needling interventions was matched (eight sessions over 2 weeks for both groups); and 5. the total number of acupoints treated per session and cumulatively over the entire course is clearly reported above to allow for dose–response considerations in future studies. Despite these efforts, we acknowledge that the two groups inevitably differed in practitioner expertise, number of treatment points per session (ten in the acupuncture group versus two in the needle-knife group), stimulation intensity, tissue injury extent, session duration (40 min versus 15–20 min), and patient–practitioner contact time, which may constitute additional sources of bias and should be considered when interpreting the results.

### Primary outcome measure

1. The VAS score was used to evaluate the pain intensity. This scale, ranging from 0 to 10, defines 0 as “no pain” and 10 as “the worst pain imaginable.” During assessment, patients were presented with the unmarked side of the scale and asked to slide the cursor to a position corresponding to their perceived pain level. The physician then recorded the score based on the cursor’s position. 2. The ODI score was employed to evaluate functional impairment associated with LDH. Scores were classified as follows: 0–20% Mild dysfunction: daily activities largely maintained; 21–40% Moderate disability: some activities require assistance; 41–60% Severe disability: daily life significantly affected, often requiring medical intervention; 61–80% Crippling disability: most activities cannot be performed; 81–100% Complete disability: bedbound or fully dependent on others. VAS and ODI scores were evaluated at baseline, post-treatment, and at the 3-month follow-up.

### Secondary outcome measures

1. Lumbar Functional Mobility (LFM): A digital protractor was used to measure changes in lumbar flexion and bilateral lateral flexion in both groups before treatment, after one course of treatment, and at a 3-month follow-up to evaluate clinical efficacy.
2. Serum levels of TNF-α, IL-1β, and IL-6 were measured in both groups before treatment and after completion of the 2-week treatment course. Blood collection and laboratory procedures: Venous blood samples were collected after an overnight fast (8–12 h fasting). Baseline blood sampling was performed within 48 h before the first intervention session; post-treatment blood collection was conducted 24 h after the final treatment session. All venous blood specimens were sent to the clinical laboratory of The Third Affiliated Hospital of Henan University of Chinese Medicine for testing. Whole-blood samples were allowed to clot at room temperature for 30 min, followed by centrifugation at 3000 r/min for 15 min to separate serum. Aliquoted serum samples were stored at −80 °C. Strictly no more than one freeze–thaw cycle was permitted prior to assay. Commercially available ELISA kits were applied for biomarker quantification. Kit manufacturer, catalog number and performance parameters were as follows: intra-assay coefficient of variation < 8%, inter-assay coefficient of variation < 12%; each sample was assayed in technical duplicate. The lower limit of detection and measurable detection range strictly followed the manufacturer-supplied kit specifications. Quantification was performed using enzyme-linked immunosorbent assay (ELISA).

### Safety assessment

All participants were monitored throughout treatment, follow-up, and each clinical visit for all predefined procedure-related adverse events (AEs), including subcutaneous hematoma, needle syncope, local infection, peripheral nerve injury, vascular trauma, and persistent post-procedural lumbago. Every newly occurring discomfort or complication was recorded in the case report form (CRF) with complete documentation covering: timing of onset, severity (mild/moderate/severe), causality assessment (definite/probable/possible/unrelated per clinical trial AE criteria), on-site management measures, and final resolution outcomes. 1. Severity grading criteria: Mild: Transient discomfort requiring no additional intervention, full spontaneous relief within 24 h; Moderate: Symptom requiring simple symptomatic management, complete resolution within 7 days without sequelae; Severe: Severe tissue injury, nerve damage, or infection requiring specialist intervention, with residual dysfunction risk. 2. Causality judgment standard: Events directly occurring during or within 24 h after intervention were classified as definite/probable treatment-related adverse events; delayed discomfort with clear alternative inducements was judged unrelated. 3. Statistical analysis for safety indicators: The incidence of adverse events (number of participants with ≥1 AE/ total per-protocol sample ×100%) was calculated for each group. Between-group comparison of AE incidence was performed using Fisher’s exact test due to small event counts. All predefined potential risks (infection, neurovascular injury, persistent pain) were actively screened at every assessment timepoint to avoid missing complications.

### Sample size

Sample size calculation was strictly performed based on the primary outcome of post-treatment VAS pain score, adhering to standard parallel-group randomized controlled trial design. All calculation parameters are fully defined and specified as follows: 1:1 group allocation ratio, two-tailed independent test, type I error rate *α* = 0.05, statistical power (1 − *β*) = 0.80, and a predefined minimal clinically important intergroup difference. Based on published relevant studies and our clinical preliminary data (18), the expected post-treatment VAS score was 3.11 (standard deviation, SD = 1.23) in the needle-knife experimental group and 5.06 (SD = 2.10) in the acupuncture control group. The pooled standard deviation of the two groups was adopted for variance estimation. The standard sample size formula for two-group quantitative outcome comparison was applied. According to the calculation results, 35 participants per group (70 participants in total) were required for valid complete-case statistical analysis. To compensate for potential participant dropout and ensure the robustness of both primary per-protocol analysis and secondary intention-to-treat sensitivity analysis, an additional 5.7% attrition rate was preset. Ultimately, a total of 74 eligible participants were enrolled in the trial.

### Statistical analysis

Data processing and analysis were conducted using IBM SPSS Statistics 26 software. Normally distributed continuous variables were presented as mean ± standard deviation (x̄ ± s); between-group comparisons were performed using independent-samples *t*-tests, and within-group comparisons were analyzed via paired-samples *t*-tests. Non-normally distributed data were expressed as median [M (P25, P75)] and analyzed using the Wilcoxon signed-rank test or Friedman test. Categorical data were analyzed using the chi-square test. Between-group differences are reported as mean difference (MD) or risk difference (RD) with corresponding 95% confidence intervals (CIs) and exact *p*-values.

Two analytical populations with corresponding estimands were predefined. The primary efficacy analysis was conducted in the per-protocol (PP) analysis population, restricted to participants who completed the full treatment course and all scheduled outcome assessments without major protocol violations. A total of 74 patients were randomized (37 patients per group); 2 patients in the NKT group and 2 patients in the AT group withdrew, leaving 35 patients in each group for the primary PP analysis. The PP analysis included only participants with complete outcome data at all scheduled timepoints; no data imputation was performed.

To evaluate the robustness of the primary findings, a pre-specified sensitivity analysis was performed on the full intention-to-treat (ITT) set, including all 74 randomized participants analyzed according to their initial random allocation, regardless of study withdrawal. For the ITT sensitivity analysis, participants were analyzed according to their initial randomization group; those with missing outcome data at any timepoint were excluded from the analysis (available-case approach).

## Results

### Patients and characteristics

Between March 15, 2025, and July 15, 2025, a total of 74 eligible patients were enrolled, including 32 females and 42 males. Among them, 70 patients (95%) completed the intervention and the 3-month follow-up, consisting of 30 females (42.8%) and 40 males (57.2%), with a mean (SD) age of 44.26 (8.35) years. The participant flow diagram is presented in Figure 4. Baseline demographic and clinical characteristics of the included patients are summarized in Table 2, with no significant intergroup differences at baseline for all indicators (all *p* > 0.05). Primary efficacy analyses were conducted in the per-protocol population (*n* = 70). Sensitivity analyses were performed in the full ITT population containing all 74 randomized participants to assess the robustness of primary findings.

**Figure 4:**
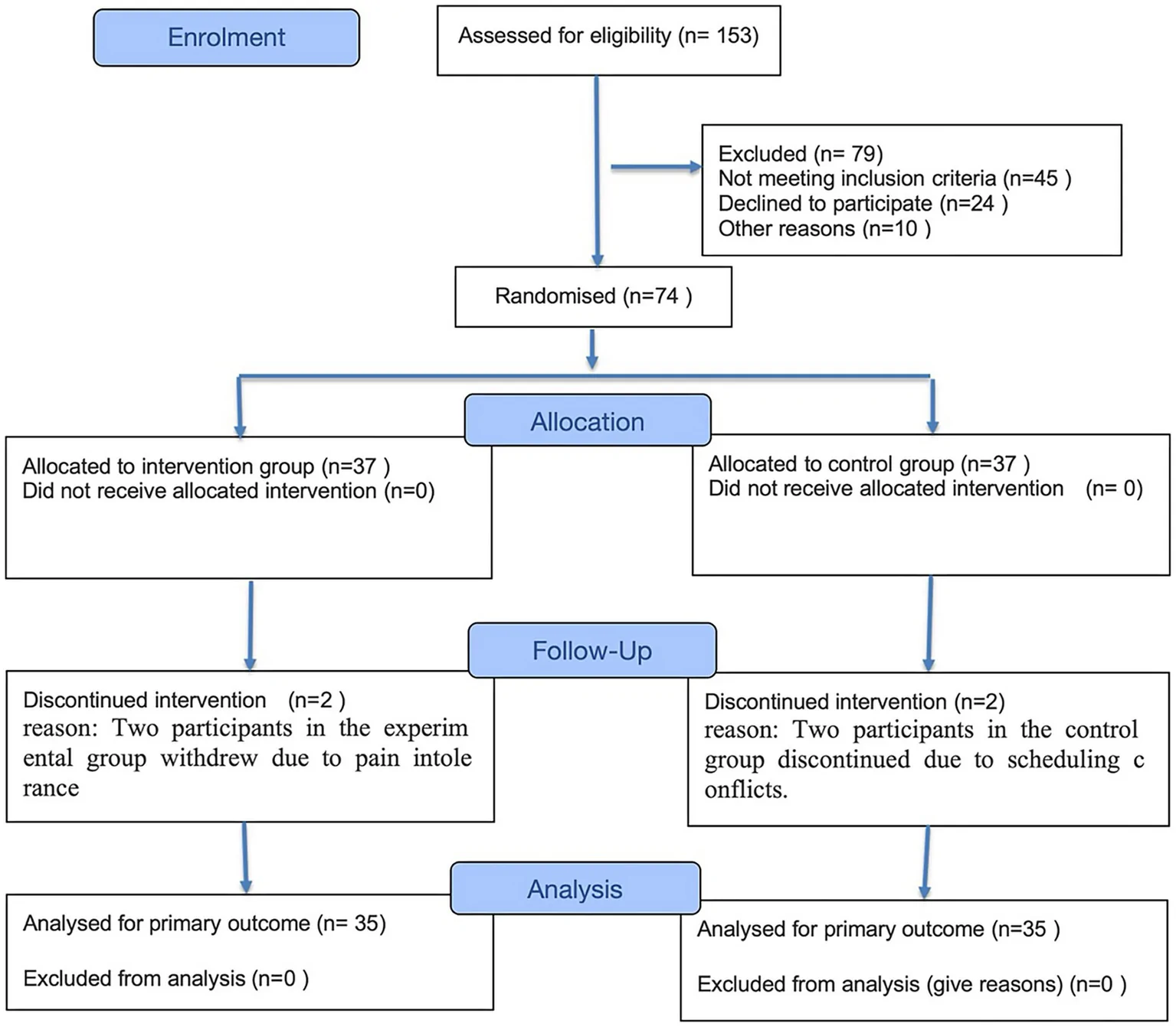
Patient inclusion process.

**Table 2:** Baseline demographic and clinical characteristics of the included participants.

Characteristic	Total sample (*n* = 74)	NKT (*n* = 37)	AT (*n* = 37)	*p* value
Female *N* (%) *χ*^2^ test (95% CI)	32 (43.2)	14 (37.8)	18 (48.6)	0.482
Male *N* (%) *χ*^2^ test (95% CI)	42 (56.8)	23(62.2)	19 (51.4)	
Smoker, *N* (%) *χ*^2^ test (95% CI)				
No	45 (60.8)	25 (67.6)	20 (54.1)	0.227
Yes	29 (39.2)	12 (32.4)	17 (45.9)	
Age, mean (SD) *t*-test (95% CI)	44.26 (8.35)	43.76 (9.23)	44.76 (7.39)	0.625
BMI, mean (SD) *t*-test (95% CI)	22.23 (14.06)	22.40 (12.41)	22.05 (15.59)	0.919
Duration of illness mean (SD) *t*-test (95% CI)	3.34 (3.05)	3.98 (3.5)	2.70 (2.5)	0.073
VAS, mean (SD) *t*-test (95% CI)	6.53 (1.67)	6.75 (1.53)	6.32 (1.80)	0.272
ODI, mean (SD) *t*-test (95% CI)	41.08 (2.87)	40.65 (3.21)	41.51 (2.43)	0.202
LFM (lumbar flexion), mean (SD) *t*-test (95% CI)	46.84 (7.28)	45.42 (8.63)	48.27 (5.30)	0.095
LFM (Left lateral flexion), mean (SD) *t*-test (95% CI)	22.56 (3.31)	21.27 (3.53)	23.85 (3.10)	0.001
LFM (right lateral flexion), mean (SD) *t*-test (95% CI)	23.57 (3.0)	23.51 (2.15)	23.63 (3.67)	0.865
Blood serum levels of, TNF - α/ng ·L mean (SD) *t*-test (95% CI)	8.87 (1.51)	8.54 (1.35)	9.21 (1.60)	0.05
Blood serum levels of, IL - 6/ng ·L mean (SD) *t*-test (95% CI)	14.01 (2.16)	14.51 (2.23)	13.50 (2.09)	0.04
Blood serum levels of, IL - 1β/ng ·L mean (SD) *t*-test (95% CI)	6.51 (0.42)	6.60 (0.50)	6.43 (0.31)	0.08

NKT, Needle knife therapy; AT, Acupuncture therapy; VAS, Visual Analogue Scale; ODI, Oswestry Disability Index; LFM, Lumbar Functional Mobility; BMI, Body Mass Index.

The ITT group-by-time interaction estimates for VAS and ODI remained significant under the Bonferroni-adjusted threshold (*α* = 0.025): VAS interaction estimate = 0.48 (95% CI: 0.17–0.79), adjusted *p* = 0.002; ODI interaction estimate = 3.05 (95% CI: 1.82–4.28), df = 70, adjusted *p* < 0.0001. The corresponding model-based between-group differences at post-treatment were VAS: −1.08 (95% CI: −1.72 to −0.44), adjusted *p* = 0.001; and ODI: −3.62 (95% CI: −4.81 to −2.43), adjusted *p* < 0.0001. At the 3-month follow-up, ITT estimates were VAS: −0.39 (95% CI: −0.65 to −0.13), adjusted *p* = 0.003; and ODI: −1.78 (95% CI: −2.48 to −1.08), adjusted *p* < 0.0001. These sensitivity findings support the robustness of the primary PP analyses, with no material changes in effect estimates or statistical inferences.”

### Primary outcomes: VAS and ODI

Table 3 presents descriptive statistics and mixed-effects model estimates for VAS and ODI scores at post-treatment and 3-month follow-up. Following the 2-week treatment cycle, the NKT group exhibited a mean (SD) VAS score of 2.25 (1.38) [95% CI: 1.78–2.72] and an ODI score of 16.35 (2.55) [95% CI: 15.48–17.22]. The AT group demonstrated a mean (SD) post-treatment VAS score of 3.41 (1.22) [95% CI: 2.99–3.83] and an ODI score of 20.13 (2.18) [95% CI: 19.38–20.88]. Both groups exhibited significant within-group improvements from baseline across all time points (all *p* < 0.001).

**Table 3:** Longitudinal outcomes of lumbosacral integrated needle-knife therapy (NKT) versus conventional acupuncture (AT).^a^

Outcome	Time point	NKT group Mean (SD) [95% CI]	AT group Mean (SD) [95% CI]	Group × time interaction (mixed model) estimate (95% CI), *p*-value^b^^,^^c^	Model-based group difference (NKT − AT) estimate (95% CI), *p*-value^d^
VAS^g^	Post-treatment	2.25 (1.38) [1.78–2.72]	3.41 (1.22) [2.99–3.83]	0.52 (0.21–0.83), ***p* = 0.001**^f^	−1.16 (−1.78 to −0.54), *p* = 0.0004
VAS	Follow-up	1.93 (0.55) [1.74–2.12]	2.36 (0.48) [2.19–2.53]		−0.43 (−0.68 to −0.18), *p* = 0.001
ODI	Post-treatment	16.35 (2.55) [15.48–17.22]	20.13 (2.18) [19.38–20.88]	3.12 (1.89–4.35), ***p* < 0.0001**	−3.78 (−4.95 to −2.61), *p* < 0.0001
ODI	Follow-up	13.17 (1.72) [12.59–13.75]	15.06 (1.29) [14.62–15.50]		−1.89 (−2.56 to −1.22), *p* < 0.0001
LFM (Flexion)	Post-treatment	75.39 (5.36) [73.55–77.23]	72.58 (5.48) [70.70–74.46]	2.81 (0.62–5.00), ***p* = 0.012**	2.81 (0.17–5.45), *p* = 0.034
LFM (Flexion)	Follow-up	79.16 (2.57) [78.27–80.05]	76.21 (2.13) [75.47–76.95]		2.95 (1.82–4.08), *p* < 0.0001
LFM (Left lateral)	Post-treatment	26.35 (3.18) [25.26–27.44]	25.65 (2.45) [24.81–26.49]	0.70 (−1.02 to 2.42), ***p* = 0.423**	0.70 (−0.70 to 2.10), *p* = 0.307
LFM (Left lateral)	Follow-up	27.67 (3.38) [26.50–28.84]	26.92 (2.79) [25.96–27.88]		0.75 (−0.75 to 2.25), *p* = 0.328
LFM (Right lateral)	Post-treatment	26.74 (2.95) [25.73–27.75]	26.33 (2.71) [25.40–27.26]	0.41 (−1.10 to 1.92), ***p* = 0.594**	0.41 (−0.92 to 1.74), *p* = 0.545
LFM (Right lateral)	Follow-up	28.05 (2.34) [27.24–28.86]	27.65 (2.28) [26.87–28.43]		0.40 (−0.70 to 1.50), *p* = 0.475
TNF-α/ng L^e^	Post-treatment	3.75 (0.60) [3.54–3.96]	3.93 (0.82) [3.65–4.21]	***p* = 0.326**	−0.18 (−0.52 to 0.16), *p* = 0.30
IL-6/ng L	Post-treatment	3.35 (0.47) [3.19–3.51]	3.82 (0.75) [3.56–4.08]	***p* = 0.028**	−0.47 (−0.89 to −0.05), *p* = 0.03
IL-1β/ng L	Post-treatment	3.52 (0.48) [3.35–3.69]	3.65 (0.53) [3.47–3.83]	***p* = 0.287**	−0.13 (−0.37 to 0.11), *p* = 0.28

^a^Data are presented as mean (standard deviation) [95% confidence interval].

^b^All estimates and *p*-values are derived from baseline-adjusted linear mixed-effects models with fixed effects for Group, Time, Group × Time, and baseline outcome value; random intercept for participant; unstructured covariance; Satterthwaite degrees of freedom.

^c^Group × time interaction estimates are presented as estimate (95% CI), degrees of freedom, *p*-value.

^d^Group difference columns present model-based contrast estimates (NKT minus AT) at each time point from the same mixed model, not from separate *t*-tests.

^e^For biomarkers (TNF-α, IL-6, IL-1β), only post-treatment measurements were available; Group × Time interaction is derived from the same model without time factor.

^f^Bold *p*-values indicate statistical significance at *p* < 0.05.

^g^Within-group changes from baseline (not shown) were all significant (*p* < 0.001) except for left and right lateral flexion in the AT group (*p* > 0.05).

Each group included 35 participants. Repeated indicators (VAS, ODI, LFM) were analyzed via baseline-adjusted linear mixed-effects models; inflammatory cytokines were analyzed by independent-samples *t*-test.

For the prespecified group-by-time interaction analyses under Bonferroni correction (*α* = 0.025), the NKT group demonstrated significantly greater longitudinal improvements than the AT group for both primary outcomes. The VAS group-by-time interaction estimate was 0.52 (95% CI: 0.21–0.83), df = 68, adjusted *p* = 0.001, and the ODI interaction estimate was 3.12 (95% CI: 1.89–4.35), df = 68, adjusted *p* < 0.0001. At post-treatment, the model-based between-group differences (NKT minus AT) were VAS: −1.16 (95% CI: −1.78 to −0.54), adjusted *p* = 0.0004; and ODI: −3.78 (95% CI: −4.95 to −2.61), adjusted *p* < 0.0001. These differences met the Bonferroni-adjusted threshold of *α* = 0.025, thereby confirming the single directional primary hypothesis with respect to immediate post-treatment superiority.

At the 3-month follow-up, both groups demonstrated further within-group improvements in VAS and ODI scores relative to post-treatment values, with the NKT group maintaining greater improvement. The model-based between-group differences at follow-up were VAS: −0.43 (95% CI: −0.68 to −0.18), adjusted *p* = 0.001; and ODI: −1.89 (95% CI: −2.56 to −1.22), adjusted *p* < 0.0001. Both sustained differences remained below the Bonferroni-adjusted significance threshold, indicating maintained superiority of NKT therapy over conventional acupuncture at 3 months. Longitudinal trajectories of these outcome measures are illustrated in Figure 5.

**Figure 5:**
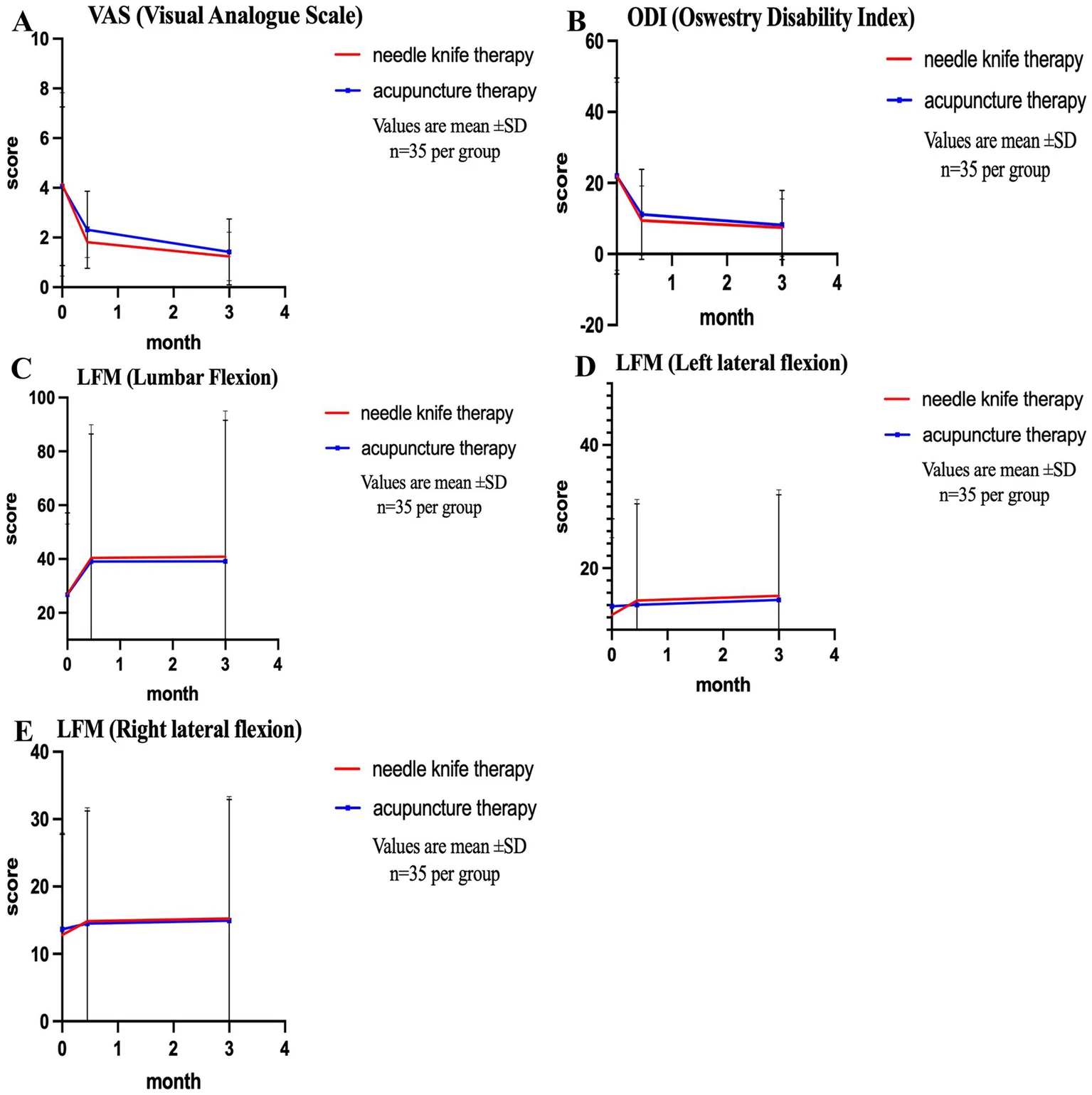
Graph showing changes in primary and secondary outcomes before and after treatment. **(A)** Visual Analogue Scale (VAS); **(B)** Oswestry Disability Index (ODI); **(C)** Lumbar Flexion (LFM); **(D)** Left lateral flexion (LFM); **(E)** Right lateral flexion (LFM).

### Secondary outcomes

#### Lumbar flexion mobility (exploratory analyses)

Improvements in all three LFM components were observed in both groups following the intervention. The NKT group demonstrated numerically higher scores compared with the AT group across all components at post-treatment (Table 3). The group-by-time interaction estimates derived from the mixed-effects model for LFM subindices were as follows: lumbar flexion estimate = 2.81 (95% CI: 0.62–5.00), *p* = 0.034; left lateral flexion estimate = 0.70 (95% CI: −1.02 to 2.42), *p* = 0.423; and right lateral flexion estimate = 0.41 (95% CI: −1.10 to 1.92), *p* = 0.594. Although the lumbar flexion interaction yielded a nominal *p*-value below the conventional 0.05 threshold, it is imperative to emphasize that these analyses were prespecified as exploratory secondary outcomes, and no adjustment for multiple comparisons was applied. Accordingly, none of the LFM interaction effects can be interpreted as providing statistically robust evidence of between-group differences, and all findings should be viewed as descriptive and hypothesis-generating.

At the 3-month follow-up, both groups exhibited further improvements in LFM relative to post-treatment measurements, with the NKT group showing more favorable numerical recovery. The model-based between-group differences at follow-up were: lumbar flexion 2.95 (95% CI: 1.82–4.08), *p* < 0.0001; left lateral flexion 0.75 (95% CI: −0.75 to 2.25), *p* = 0.328; and right lateral flexion 0.40 (95% CI: −0.70 to 1.50), *p* = 0.475. In keeping with the exploratory framework, no formal inferential conclusions regarding LFM superiority were drawn from these analyses.

#### Serum inflammatory cytokines (exploratory analyses)

Both groups exhibited reductions in serum inflammatory cytokine levels following treatment, with the NKT group demonstrating greater numerical decreases than the AT group at post-treatment for all three markers. As inflammatory cytokines (TNF-α, IL-6, and IL-1β) were assessed exclusively at baseline and post-treatment—with no 3-month follow-up measurements—repeated-measures mixed-effects models were inapplicable. Instead, between-group comparisons were performed using independent-samples *t*-tests with baseline adjustment, applying an unadjusted significance threshold of *α* = 0.05.

The post-treatment between-group differences (NKT minus AT) were as follows: TNF-α difference = −0.18 ng·L (95% CI: −0.52 to 0.16), unadjusted *p* = 0.30; IL-1β difference = −0.13 ng·L (95% CI: −0.37 to 0.11), unadjusted *p* = 0.28; and IL-6 difference = −0.47 ng·L (95% CI: −0.89 to −0.05), unadjusted *p* = 0.03. Only IL-6 showed a nominally significant unadjusted between-group difference. Nevertheless, given the exploratory nature of these biomarker analyses and the absence of multiplicity adjustment, this isolated finding should be interpreted with considerable caution and regarded as hypothesis-generating rather than confirmatory.

#### Adverse event

All predefined adverse events including subcutaneous hematoma, needle syncope, skin infection, neurovascular injury and persistent post-procedural pain were closely monitored among the 70 per-protocol participants (35 in the NKT group and 35 in the AT group); no cases of infection, nerve injury, vascular trauma or persistent postoperative pain were detected during the entire trial period. In the needle-knife therapy group, only one participant suffered mild subcutaneous hematoma with an incidence rate of 2.86% (1/35), which was judged probably related to lidocaine injection and needle-knife puncture; local circular massage was administered right after discovery, and the hematoma was fully absorbed within 48 h without residual bruising or discomfort. Two participants in the conventional acupuncture group experienced independent mild needle syncope during their first treatment session, corresponding to an incidence rate of 5.71% (2/35), which was definitely triggered by acupuncture stimulation and autonomic reflex; we placed the patients supine with elevated lower limbs, provided warm sugar water and continuously monitored vital signs, and all symptoms such as dizziness and nausea were completely relieved within 5 min with no recurrent syncope in subsequent sessions. Fisher’s exact test showed no significant intergroup difference in the overall incidence of adverse events, and no moderate or severe adverse reactions occurred in either group.

## Discussion

This single-center randomized controlled trial demonstrated that 14-day needle-knife lumbosacral therapy induced distinct longitudinal improvement trajectories in VAS and ODI compared with acupuncture treatment. Favourable clinical improvements in VAS and ODI were maintained up to the 3-month time point within the trial’s observation window. Caution should be exercised when extrapolating outcomes to longer follow-up durations beyond 3 months. No severe adverse events occurred in either group, and intergroup adverse event incidence showed no statistical difference, confirming the acceptable safety profile of both interventions under standardized operation.

In this study, the within-group changes with a large effect size in pain intensity exceeded the minimal clinically important difference (> 10%) for VAS, indicating clinically meaningful improvements (19). These results suggest that needle knife lumbosacral therapy effectively reduces localized pain in LDH patients, with sustained therapeutic effect throughout the 3-month period. The similar findings have been found in the previous studies regarding the positive role of the needle-knife therapy. A single-blind randomized controlled trial has demonstrated that needle-knife therapy provides more significant short- and long-term pain reduction and recovery of local dysfunction compared with acupuncture therapy (20). Compared with conventional needle-knife protocols for lumbar disc herniation (LDH), which primarily target the release of tension in paravertebral soft tissues—such as decompression at the L3–L5 transverse processes (21) or ligamentum flavum release (22), this study employed a standardized, integrated lumbosacral soft tissue release protocol. This approach encompassed both lumbosacral and sacroiliac structures as a unified therapeutic package. Within our single-center cohort, we generated exploratory evidence comparing this multimodal needle-knife intervention against routine acupuncture.

Notably, the needle-knife group demonstrated superior longitudinal improvements in VAS scores and ODI. These findings were supported by significant group-by-time interaction effects that met the prespecified Bonferroni-adjusted significance threshold (*α* = 0.017). Consequently, this trial provides exploratory evidence supporting the favorable efficacy of the integrated lumbosacral needle-knife package relative to routine acupuncture. However, it does not establish superiority over standard conventional needle-knife protocols.

Although numerically greater gains in LFM were observed in the needle-knife group, most group-by-time interaction estimates for LFM failed to reach the predefined adjusted significance threshold following multiple testing correction. Therefore, these descriptive numerical differences in lumbar mobility cannot be interpreted as statistically reliable evidence of superior functional recovery. Furthermore, the observed intergroup outcome differences cannot be exclusively attributed to sacroiliac joint release or the independent mechanical cutting action of the needle-knife instrument. To delineate the specific therapeutic contribution of sacroiliac-targeted release, formal head-to-head randomized controlled trials directly comparing the full lumbosacral needle-knife regimen with conventional paravertebral-only needle-knife therapy are warranted.

The therapeutic mechanisms underlying needle-knife intervention in LDH are hypothesized to operate through four interconnected pathways. Structurally, it is proposed that precise percutaneous release of paravertebral soft tissue constraints alleviates mechanical nerve root compression by disrupting adhesive scar tissue and myofascial contractures (23). At the cellular level, prior literature suggests that mechanical stimulation of nociceptive fibers may trigger TRPV1 channel opening, initiating intracellular calcium influx that subsequently dampens NF-κB nuclear translocation and downstream pro-inflammatory gene transcription (24). Although this pathway is theoretically associated with reduced circulating IL-6 and TNF-α levels, the current study did not demonstrate consistent between-group differences across all measured cytokines. Therefore, these biomarker findings should be interpreted as clinical associations rather than definitive evidence of causal molecular modulation. Biomechanically, capsular release of zygapophyseal joints and intertransverse ligament mobilization is thought to correct segmental rotational dysfunction and pelvic obliquity, thereby reestablishing lumbopelvic-lower extremity kinetic chain alignment (25). Neurochemically, paraspinal afferent stimulation may engage spinal cord μ-opioid receptor populations and precipitate β-endorphin secretion, potentially generating prolonged antinociception (26) (Figure 6). However, these neurochemical changes were not directly assessed in this trial, and the reported clinical outcomes cannot be construed as proof of structural decompression, specific molecular signaling activation, or dorsal root ganglion modulation. Consequently, the proposed mechanisms remain hypotheses derived from existing rationale, distinct from the empirical mechanistic evidence provided by the measured cytokine profiles in this study.

**Figure 6:**
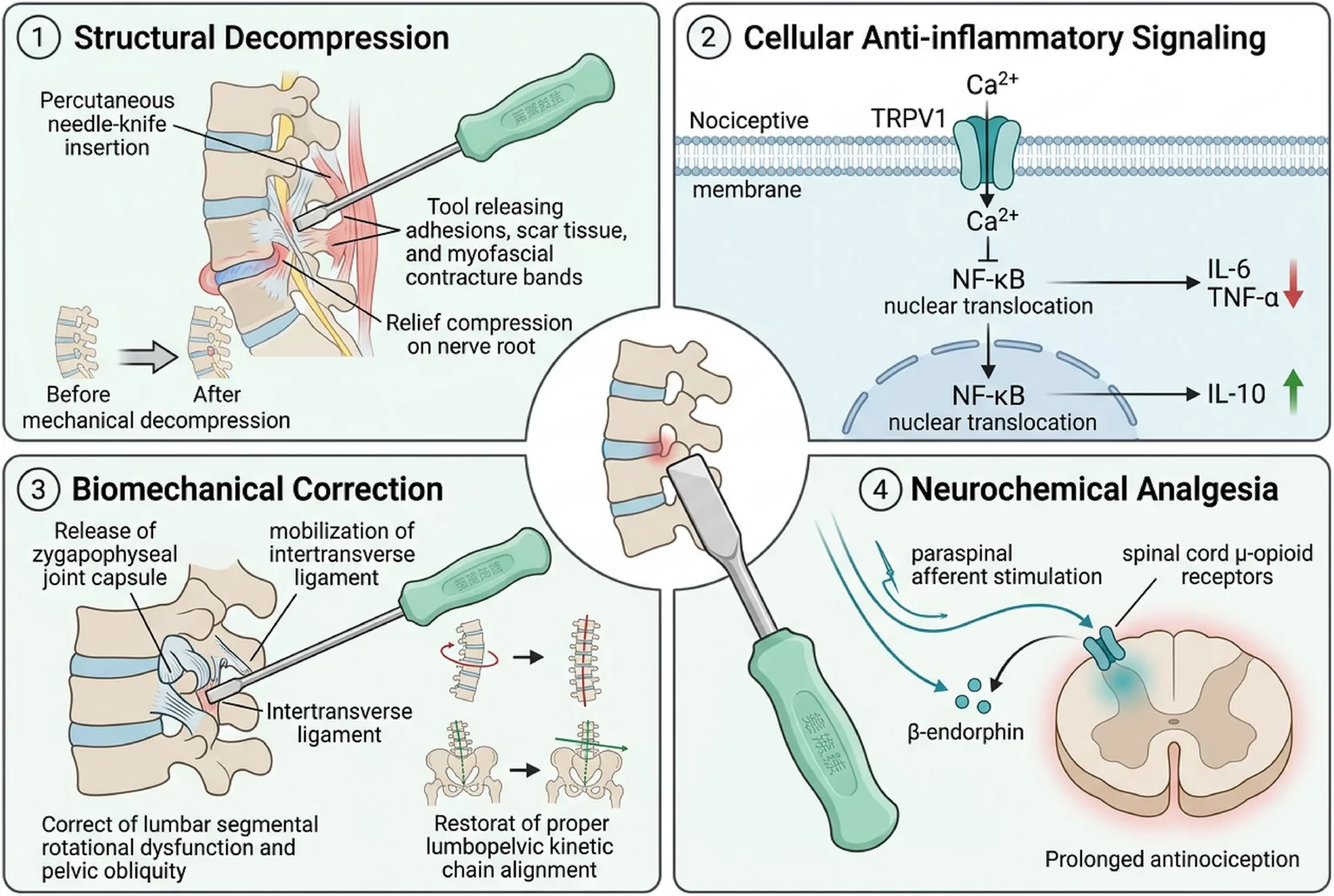
Mechanisms of needle-knife therapy for the treatment of LDH.

LDH is closely related to chronic soft tissue injuries (27). In the early stage of LDH, it mostly presents as multifidus muscle atrophy and adhesion of the thoracolumbar fascia, resulting in instability of the lumbar segments, microcracks in the annulus fibrosus, and gradually accumulating micro-damage (28). Subsequently, the sacrospinous ligament and the gluteus maximus undergo compensatory hypertrophy to maintain the stability of the lumbar segments, and the additional tension is continuously transmitted through the fascial chain to the intervertebral disc (29). Once the compensatory muscle groups become fatigued or form scar contractures, the accumulated stress in the intervertebral disc will exceed the degeneration threshold, thereby causing the nucleus pulposus to protrude (30). Therefore, during the treatment, needle-knife therapy emphasizes the release of tension in soft tissues surrounding the spine to restore the dynamic biomechanical balance of the spine (31).

Based on extensive clinical experience, our team has proposed the “Iceberg Theory” (32) of chronic soft tissue injury (Figure 7). We believe that most chronic soft tissue injuries evolve gradually. As the patient’s physiology adapts to the localized pathological changes, the clinical presentation becomes less prominent. Consequently, chronic soft tissue injury can be divided into visible and hidden parts. The “tip of the iceberg” signifies the most symptomatic region—the area eliciting the greatest discomfort or functional impairment—and is readily apparent to clinicians. Subclinical soft tissue injuries are submerged “beneath the waterline” and may be vaguely perceived or entirely unnoticed by the patient and often missed by physicians. These concealed injuries represent the continuous progression of the disease. For diseases related to soft tissue injuries, the covert component is the primary target of clinical intervention. Just as “the center of gravity of an iceberg lies below the water level rather than above it,” the core pathogenesis often lies beyond the patient’s overt symptoms. This necessitates a focus beyond superficial manifestations to address the root cause of the disease, identifying the true source of the patient’s pain.

**Figure 7:**
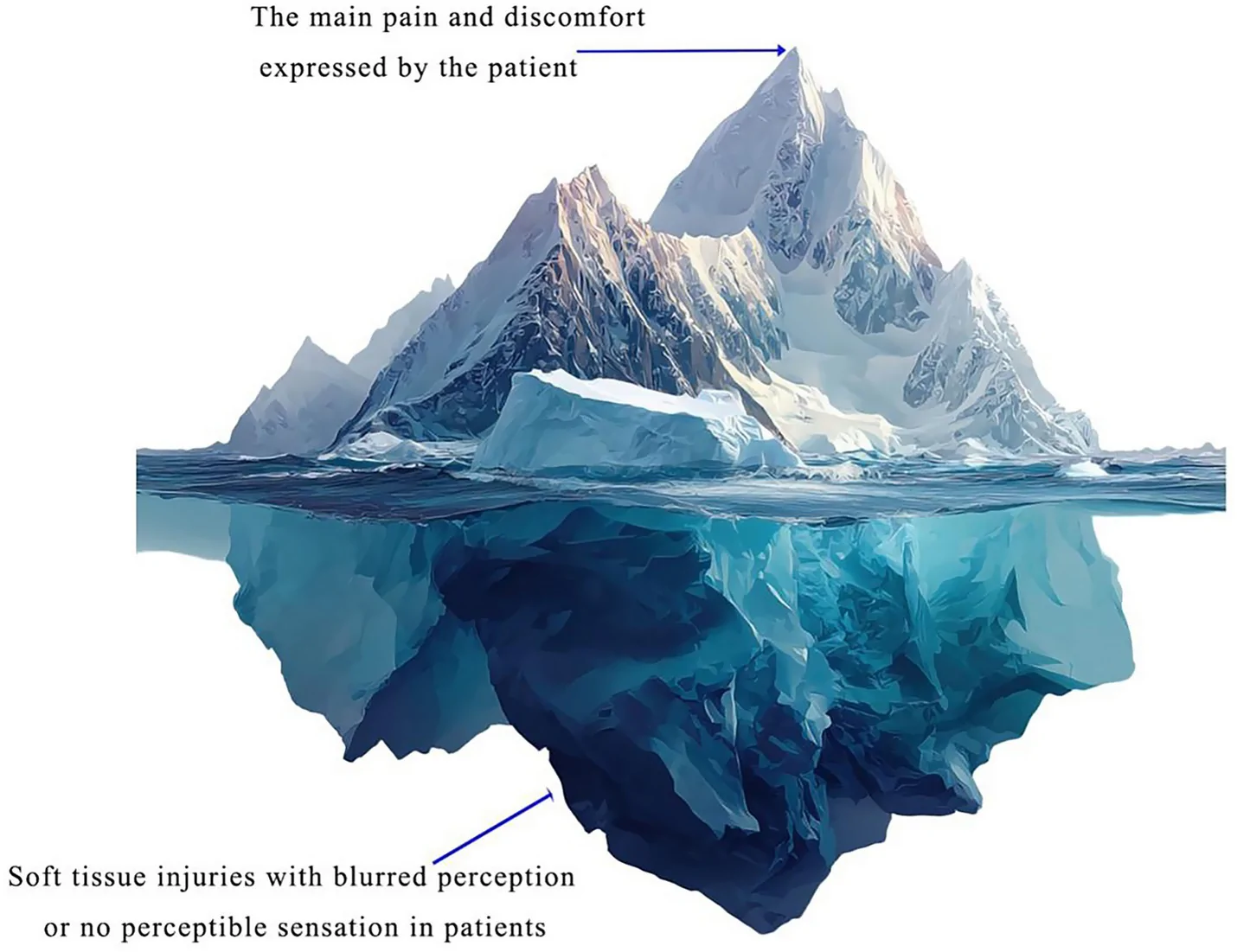
Iceberg theory. The “tip of the iceberg” signifies the most symptomatic region—the area eliciting the greatest discomfort or functional impairment—and is readily apparent to clinicians. Submerged “beneath the waterline” are subclinical soft tissue injuries, which may be vaguely perceived or entirely unnoticed by the patient and often missed by physicians.

The “Needle-knife lumbosacral therapy” is established based on this rationale. The sacral dorsal root ganglion (DRG) is a key sensitization center for chronic low back pain and lower extremity radicular pain (33). Chronic inflammatory or mechanical stimulation of the surrounding soft tissues, including the multifidus muscle, sacrospinalis muscle, and sacroiliac ligament, can result in sustained DRG activation, contributing to the chronicity and refractoriness of low back pain symptoms (34). Consequently, soft tissue release in the sacral region can reduce mechanical sensitization of the DRG, thereby mitigating low back pain symptoms.

This study has several strengths. The use of blinding in the trial design and execution, which improved the reliability and validity of the results. Standardized training for practitioners before the intervention, along with standardization for manipulation techniques and needling depth, minimized operator-dependent variability and enhanced the evidence’s quality. Moreover, a comprehensive battery of outcome measures, such as pain scores, dysfunction assessments, range of motion, and inflammatory markers, enabled a thorough evaluation of the needle-knife’s clinical efficacy in treating LDH.

However, several methodological limitations warrant consideration. First, the inclusion criteria relied primarily on clinical symptoms (low back pain with radiating leg pain) without requiring CT or MRI confirmation of lumbar disc herniation or nerve root compression, nor did the manuscript systematically report neurological examination findings, straight-leg-raising test results, sensory deficits, or muscle weakness. This diagnostic approach may have failed to adequately exclude other conditions that present with similar symptomatology, including lumbar muscle strain, lumbar spinal stenosis, spondylolisthesis, hip disorders, sacroiliac joint disorders, and nonspecific low back pain, potentially leading to diagnostic misclassification and thereby compromising internal validity and the interpretability of the study findings. Second, local infiltration anesthesia (1 mL of 0.5% lidocaine per site) was administered exclusively in the needle-knife group, whereas the acupuncture group received neither anesthetic nor sham injection; this design was adopted for ethical and safety reasons—needle-knife manipulation entails deep soft tissue penetration down to the periosteum, and anesthesia is essential to ensure patient tolerance and procedural safety—while also reflecting routine clinical practice and enhancing ecological validity. Nevertheless, this asymmetry introduces a substantial confounding effect, as between-group differences incorporate not only the distinct therapeutic modalities but also the analgesic effect of lidocaine, the physiological and psychological responses to injection, and differential procedural exposure. To disentangle these effects, we reason that the short half-life of lidocaine (approximately 1.5–2 h) cannot explain the sustained between-group differences observed at 24–48 h post-treatment or, more compellingly, at the 3-month follow-up (VAS: *p* = 0.001; ODI: *p* = 0.0001). Moreover, the greater reduction in serum IL-6 levels in the needle-knife group (*p* = 0.03) provides objective biomarker evidence supporting a specific therapeutic effect beyond transient anesthesia, implicating mechanical release of soft tissue adhesions and modulation of the local inflammatory microenvironment as plausible mediators. Despite this reasoning, we acknowledge that the confounding influence of lidocaine on immediate post-treatment subjective outcomes—particularly VAS scores—cannot be definitively excluded, constituting a major source of bias in this trial. Third, the restricted generalizability of findings beyond the studied chronic moderate-to-severe LDH population due to the single-center design and stringent inclusion criteria limits external validity. Fourth, incomplete blinding of participants and practitioners may introduce expectation, performance, and reporting biases, although outcome assessors remained blinded. Fifth, the 3-month follow-up period is insufficient to assess long-term durability and recurrence. Finally, the modest sample size (*n* = 70 per-protocol) may have underpowered detection of intergroup differences for several secondary outcomes (e.g., lateral flexion, TNF-α, IL-1β) despite observable clinical improvement trends. Recognition of these limitations is essential for balanced interpretation, and future trials should incorporate mandatory imaging confirmation for diagnosis, sham-controlled designs with unified anesthesia protocols, larger sample sizes, and extended follow-up to definitively validate the efficacy of needle-knife lumbosacral therapy for LDH.

## Conclusion

In conclusion, significant between-group differences in longitudinal changing trajectories of VAS and ODI were detected during the 3-month follow-up. For LFM and inflammatory cytokines evaluated in this trial, available data provided inconclusive evidence to support intergroup superiority. Favourable outcomes persisted until the 3-month assessment within the current observation window. Further research with prolonged follow-up and larger sample sizes is needed to verify sustained therapeutic effects of integrated lumbosacral needle-knife therapy.

## Data Availability

The raw data supporting the conclusions of this article will be made available by the authors, without undue reservation.
