# Supplementary material for: Needle-knife lumbosacral therapy for lumbar disc herniation: a single-center, outcome assessor-blinded randomized controlled trial
Source: Front Neurol. 2026 Oct 16;17:1898714. doi: 10.3389/fneur.2026.1898714 (PMC13719306; doi:10.3389/fneur.2026.1898714)
Supplement: Supplementary file 1 [file Supplementary_file_1.docx]

**Supplement1**

**Ethics approval and consent to participate**

This prospective outcome assessor-blinded randomized controlled trial was conducted as part of the research project titled Clinical Efficacy Analysis of Chronic Nonspecific Low Back Pain Treated by "Occasional Stabbing Method" with Needle Knife. The trial protocol was reviewed and approved by the Institutional Review Board (IRB) of The Third Affiliated Hospital of Henan University of Chinese Medicine (Approval No.: 2025HL-003; approval date: February 27, 2025).The trial was pre-registered on the International Traditional Medicine Clinical Trial Registration Platform (Registration ID: ITMCTR2025002191) prior to enrollment of the first participant. The registration completion date was March 10, 2025, while the first eligible patient was recruited on March 15, 2025, complying with the requirement of prospective trial registration. The registration webpage link: [https://itmctr.ccebtcm.org.cn/mgt/project/user/approved-list](https://itmctr.ccebtcm.org.cn/mgt/project/user/approved-list). All participants provided written informed consent before trial enrollment in accordance with the Declaration of Helsinki. Study staff fully explained trial objectives, intervention schedules, potential benefits, foreseeable risks, alternative therapies, and participants’ rights and obligations to each candidate participant. Subjects were only enrolled after voluntary signature of the informed consent document.If new ethical considerations arise during trial implementation, the investigators will submit written protocol amendments to the IRB for re-approval; renewed informed consent will be obtained from all enrolled participants after the amendment is authorized. For participants who voluntarily withdraw from the trial, all valid de-identified data collected before withdrawal will be retained for per-protocol and intention-to-treat statistical analyses.

Protocol Registration vs Final Manuscript Consistency Comparison

We systematically cross-compared the pre-registered trial protocol, pre-specified statistical analysis plan (SAP), and the final submitted manuscript on core trial elements, with full consistency demonstrated as follows:

Primary outcomes: Pre-registered primary endpoints were Visual Analog Scale (VAS) and Oswestry Disability Index (ODI), consistent with the final manuscript. Assessment timepoints (baseline, post 2-week treatment, 3-month follow-up) remained unchanged.

Secondary outcomes: Pre-specified secondary indicators included lumbar functional mobility (LFM) and serum inflammatory cytokines (TNF-α, IL-1β, IL-6), identical to the indicators reported in the final text. Cytokines were scheduled for detection only at baseline and post-treatment without long-term retesting, matching the actual trial operation.

Sample size calculation: The registered SAP calculated a required sample of 35 subjects per group (70 total) based on prior clinical data; 74 participants were enrolled to account for potential dropout, consistent with the manuscript’s sample design.

Follow-up duration: The registered protocol specified a 3-month post-treatment follow-up window, which was strictly implemented without extension or shortening in the trial.

Statistical methods: The pre-registered statistical plan pre-defined independent/paired t-tests, χ² test, mixed-effects linear models for repeated measurements, Bonferroni multiple testing correction, per-protocol (PP) primary analysis, and supplementary intention-to-treat (ITT) analysis with multiple imputation. All analytical strategies described in the manuscript fully conform to the pre-registered SAP.

**Protocol Amendments Disclosure**

Only one formal protocol amendment was submitted to and approved by the IRB, with full details disclosed below:

**Amendment Version: V2.1**

Issue Date of the revised protocol: December 06, 2025

Rationale for amendment: The original registered protocol only specified per-protocol analysis as the sole analytical strategy. After completing raw data collection but prior to unblinding and statistical examination of trial outcomes, the research team supplemented intention-to-treat (ITT) analysis and multi-dimensional sensitivity analysis into the statistical plan to comply with CONSORT 2025 reporting standards for randomized controlled trials.

Timing of modification: All changes were finalized and IRB-approved before any statistical testing or outcome interpretation was performed, without post-hoc adjustment of primary/secondary endpoints, follow-up duration, or sample size targets. No other alterations to trial interventions, outcome indicators, or inclusion/exclusion criteria were made throughout the study.

**Dissemination**

Study results will first be fed back individually to all enrolled participants. Findings will be further disseminated via academic lectures, clinical training courses, and open internet resources to clinical researchers and the general public, regardless of whether the intervention shows positive, neutral or negative therapeutic effects. The complete trial dataset and analytical conclusions will be published as a peer-reviewed academic article.

Consent for publication

Not applicable. No individual patient case images or identifiable clinical records are included in the manuscript.

**Availability of data and materials**

The original submitted manuscript was accompanied by this trial protocol supplement. The full raw trial dataset, original statistical output files, informed consent templates, and IRB approval materials are available from the corresponding author upon reasonable formal request. Minor unmentioned supplementary materials can also be obtained by contacting the corresponding author via the contact information listed in the main text.Correction: Revise "More date" to "More data"

**Patient and Public Involvement**

All participants were recruited from the Acupuncture Department of The Third Affiliated Hospital of Henan University of Chinese Medicine. Patient representatives were not involved in trial design, outcome selection or data analysis; the final aggregate trial results will be released via peer-reviewed journal publication for public and clinical reference.

**Informed consent statement**

All enrolled participants provided written informed consent for participation and anonymous publication of aggregate trial data. No identifiable individual patient information will be released in any published outputs.

**Authors’ contributions**

Yiming Zhang and Ruiguo Li conceived the trial and designed the original registered protocol; Xiaohong Zhang and Zhanyou Wang drafted the initial manuscript and supplementary protocol file; Ziwei Zhang, Huanhuan Xu and Xiaofan Li conducted data collation, cross-checking between registered protocol and final text, and drafted the protocol amendment description. All authors reviewed and approved the registered protocol, amended version, and final supplementary materials.

**Competing interests**

The authors declare no competing interests.

Issue Date: 06 December, 2025

Protocol Amendment Version: 2.1

**Figure1 Ethical approval materials for research projects**

| Review date | January 14, 2025 |
| --- | --- |
| Location of review | Jinshui District, Zhengzhou City, Henan Province |
| Clinical research approvals | NA |
| Clinical Research Program | Clinical Efficacy Analysis of Chronic Nonspecific Low Back Pain Treated by "Occasional Stabbing Method" with Needle Knife |
| Review of  documentation | informed consent |
| Contract Research Organization/CRO | The Third Affiliated Hospital of Henan University of Chinese Medicine |
| Clinical Research Organization | The Third Affiliated Hospital of Henan University of Chinese Medicine |
| Principal Investigator | Li Ruiguo, Wang Zhanzong, Zhang Shuai zhou |
| Review methods | ■Conference review □Expedited review |
| Approval validity date | February 27, 2025, to February 26, 2026 |
| The Third Affiliated Hospital of Henan University of Chinese Medicine  Date : February 27,2025 | |
